# Knowledge discovery in databases of biomechanical variables: application to the sit to stand motor task

**DOI:** 10.1186/1743-0003-1-7

**Published:** 2004-10-29

**Authors:** Giuseppe Vannozzi, Ugo Della Croce, Antonina Starita, Francesco Benvenuti, Aurelio Cappozzo

**Affiliations:** 1 Department of Human Movement and Sport Sciences, University Institute for Movement Science, Roma; 2 Department of Biomedical Sciences, University of Sassari, Sassari, Italy; 3 Department of Informatics, University of Pisa, Pisa, Italy; 4 Department of Rehabilitation, AUSL 11, San Miniato, Pisa, Italy

**Keywords:** knowledge discovery, data mining, association rules, human movement, sit to stand

## Abstract

**Background:**

The interpretation of data obtained in a movement analysis laboratory is a crucial issue in clinical contexts. Collection of such data in large databases might encourage the use of modern techniques of data mining to discover additional knowledge with automated methods. In order to maximise the size of the database, simple and low-cost experimental set-ups are preferable. The aim of this study was to extract knowledge inherent in the sit-to-stand task as performed by healthy adults, by searching relationships among measured and estimated biomechanical quantities. An automated method was applied to a large amount of data stored in a database. The sit-to-stand motor task was already shown to be adequate for determining the level of individual motor ability.

**Methods:**

The technique of search for association rules was chosen to discover patterns as part of a Knowledge Discovery in Databases (KDD) process applied to a sit-to-stand motor task observed with a simple experimental set-up and analysed by means of a minimum measured input model. Selected parameters and variables of a database containing data from 110 healthy adults, of both genders and of a large range of age, performing the task were considered in the analysis.

**Results:**

A set of rules and definitions were found characterising the patterns shared by the investigated subjects. Time events of the task turned out to be highly interdependent at least in their average values, showing a high level of repeatability of the timing of the performance of the task.

**Conclusions:**

The distinctive patterns of the sit-to-stand task found in this study, associated to those that could be found in similar studies focusing on subjects with pathologies, could be used as a reference for the functional evaluation of specific subjects performing the sit-to-stand motor task.

## Background

In the last decade quantitative movement analysis has been increasingly used in clinical contexts [1]. This analysis makes use of fairly complex instrumentation and of models of the musculo-skeletal system. It provides a great amount of information, such as space and time characteristics of the motor task analysed, joint and segment kinematics and kinetics and electromyographic patterns of muscular recruitment. An integrated analysis of measured and estimated biomechanical quantities allows for the description of the subject performance, for the discrimination among different motor strategies and, therefore, it supports the clinical decision-making process [2].

Modern complex instrumentation and models, such as stereophotogrammetric systems and multi-segment models of the human body, provide a thorough and faithful description of the subject's movement at a local level (e.g. joints kinematics), to be used at its best as a support to the functional assessment of subsystems of the locomotor apparatus (e.g. joint function) [3]. However, the large amount of measured information is not paralleled by the capability of such information of supporting the assessment of the overall subject's mobility [4]. Simpler experimental set-ups and models may be more appropriate to functionally assess a subject performing a specific motor task [5]. In recent years, clinical tests have been devised aimed at quantitatively assessing the level of a subject's activity limitation based on simple and encumbrance-free experimental set-ups associated with mechanical models of the musculo-skeletal system. These models are designed to be associated with both the subject and the specific task being performed [6]. In this context, Minimum Measured Input Models (MMIM) have been proposed and proven to offer effective insights into the motor task execution [7]. Simplified, and therefore low-cost, experimental set-ups facilitate the gathering of data both locally (a shorter examination time is needed) and in multi-centre contexts (more laboratories can afford the necessary experimental set-up), allowing the collection of a great quantity of data which may be sent to a single data repository.

However, even simplified experimental setups and models may provide a large amount of biomechanical data that requires considerable human efforts to be interpreted [8]. In fact, traditional methods of data analysis for the extraction of knowledge rely on a direct analysis, which is usually demanding and time-consuming, and on the interpretation of an experienced analyst [9]. Such analysis becomes hardly applicable when dealing with data collected multi-centrically.

The aim of this study was to extract knowledge regarding the execution of a specific motor task. The term "knowledge" refers here to any relationship among attributes associated with the phenomenon under analysis. These relationships can be intended as causal and, therefore, suitable for the interpretation endeavours, or at least as tools for evidencing the presence of a repeatable pattern of variables. The declared goal was pursued by searching relationships among large amounts of biomechanical quantities by using an automatic method. Some *data mining *techniques (*data mining *is a step of a process called Knowledge Discovery in Databases (KDD)) lend themselves to be effectively used in this context since they may reveal meaningful patterns and data structures from massive databases [10,11]. A specific *data mining *technique was applied to the data yielded by the analysis of sit-to-stand (STS) trials performed by healthy adults and carried out using the above-mentioned MMIM approach. The STS motor task was chosen because it has been shown to be adequate for determining the level of subject-specific motor ability [12]. In addition, the data provided by MMIMs were shown to be powerful overall descriptors of motor tasks. A group of unrestricted age and gender healthy adults was used with the goal of discovering knowledge inherent to the way healthy adults perform the selected motor task.

In order to properly frame this study, a summary description of the MMIM approach and an overview of the KDD process are reported.

## Methods

### A MMIM applied to the STS task – The TIP model

A MMIM is a model of a portion of the musculoskeletal system that includes the invariant aspects of both the modelled mechanical system and the motor task being performed. Therefore, a MMIM requires a minimum amount of measurements and provides a physiology-related description of the motor task [4]. In analysing the STS, only measurements from a single force platform are needed. The task is divided in two time phases: before- and after-seat-off (BSO and ASO). In each time phase a Telescopic Inverted Pendulum (TIP) model is applied. A TIP is characterised by a fixed base of support and by a massless link joining the base of support of the moving portion of the body to its centre of mass (CM). The link can elongate, controlled by a linear actuator (LA), and can rotate around its base of support, controlled by two actuators acting in the sagittal (SA) and frontal (FA) plane, respectively. The kinematics of, and the dynamic actions on, the CM of the modelled portion of the body involved in the movement are needed as model inputs. The outputs of the TIPs are the kinematic and kinetic variables associated with the actuators. During BSO the TIP is applied to the upper part of the body with its base of support positioned on the chair, while during ASO the TIP is applied to the whole body and its base of support is located at the ankles. In order to apply the TIP model in each phase, subject specific and experimental set-up parameters are set. A list of TIP parameters and TIP output variables may be [7] collected into a database.

### The KDD process

**Figure 1:**
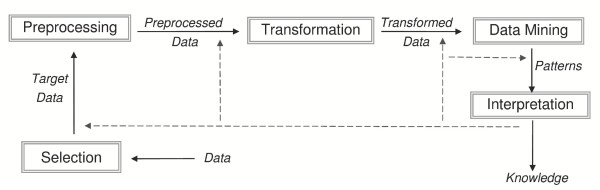
A scheme of the KDD process. Input data are initially selected and target data are isolated. Pre-processing and transformation are performed to ensure the database reliability. Data mining is the core analysis. The knowledge discovery process ends with the interpretation of the results.

The KDD process was introduced in order to provide a framework in which data-miners could work in a logical and sequential way, considering all the research aspects from the data acquisition to the information extraction [13,14]. An iterative five phase process may be adopted (Figure 1) [15].

Initially, the domain understanding, the parameter selection, and the goal definition need to be set. A subset of interest of the stored dataset can then be isolated. Pre-processing is performed to reduce noise and fill possible gaps in the target dataset. Elimination of outliers, corrections of wrong elements in the database and reduction of dimensionality are crucial transformations to reach an adequate level of suitability of the database.

*Data mining *"is a well-defined procedure that takes data as input and produces output in the form of models or patterns" [16] and is the core of the KDD process. It is used with different aims such as Exploratory Data Analysis (EDA) [17], Descriptive Modelling [18], Predictive Modelling such as Classification and Regression [19], Retrieval by Content [20] and Discovering Patterns or Rules [21].

Innovative techniques for the data mining have been introduced to be used either in conjunction with or in alternative to traditional statistical methods for two main reasons. First, while classical statistics is applied to data collected according to a specific goal of the analyst, data mining methods are applied to data already collected and aim at finding unknown relationships among them. Secondly, data mining allows to infer general rules with adequate approximation, even if the amount of data available is not as large as that generally required by inferential statistics [16].

The selection of the *data mining *technique is based on the specific analysis. Prediction, clustering, classification and research of association rules are the most common tasks and each of them may be accomplished with various algorithms. Finally, data interpretation helps the user in managing and understanding the results: visualisations (clustering) or extraction of symbolic rules are common ways of evaluating the discovered knowledge.

### The search for association rules

The technique of research of association rules, which aims at finding the most recurrent patterns in a database, was selected for the data mining. Given a database D of experimental trials *T*, each experimental trial is a record of D and is made of a set *X *of literals called items. An item represents a specific value of an attribute of a table of D, and a record can be represented as an attribute (i.e. an output variable or a model parameter) together with its value [21]. The problem may be defined as follows. Let *I *= {i_1_, i_2_,...., i_m_} a set of items of D therefore, *T *can be seen as a group of items such that *T **I*. An association rule can be defined as a logical implication:

*X **Y*

where *X **I *is the antecedent of the rule, *Y **I *is the consequent and *X *∩ *Y *= . A rule *X **Y*, over a set of trials *T*, has a confidence *c *if *c *% of the trials in *T *containing *X*, also include *Y*. The same rule in the same context has a support s if *s *% of the trials in *T *contain *X **Y*. The confidence of a rule *X **Y *can be calculated from the support of the antecedent *X *and the support of the union of the antecedent *X *and the consequent *Y*:



Confidence is an index of the validity of a rule. A high confidence means that there is a strong relationship between *X *and *Y *in the sense that the presence of a pattern *X *in a trial implies, with a high probability, the presence of *Y *in the same trial. Given a set of trials *T*, finding "interesting" association rules in *T *is the problem of generating all the rules whose both support and confidence are greater than a set threshold (*minimum support *and *minimum confidence*).

The extracted rules were reported in the following format:

*A *→ *B *[*c *% ]

where the first item was the antecedent of the rule, the item which followed was the consequent, while the value indicated in square brackets was the confidence. The implication was intended to be valid only one way, from the left to the right. In case of validity of both directions, "definitions" were obtained:

*A *←→ *B *[*c-min %*]

composed by two rules having the two items both as antecedent and consequent whose confidence *c-min *was the lowest of the two *c *values associated with the one way rules. The search for association rules followed the path illustrated in Figure 1. Initially, data were read from the sources and displayed, allowing for a straightforward selection of the dataset of interest. Next, the subset of data were prepared to the analysis by eliminating possible outliers and filling possible gaps in the database due to incorrect applications of the model. Typically, data preparation is the most time consuming phase of the KDD, but is also highly crucial since the effectiveness of a data mining analysis relies on the consistency of the database.

**Figure 2:**
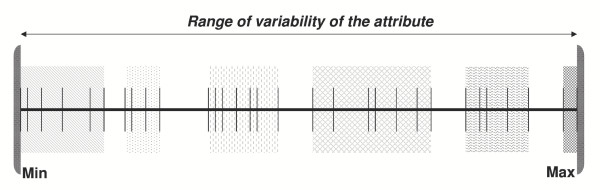
Example of a discretisation process of a quantitative attribute. Grey areas represent the different partitions, i.e. the items. Vertical lines represent the values of the quantitative attribute.

Since the theory of association rules was formulated to deal with qualitative attributes [22] characterised by a limited number of scores, the virtually infinite values of quantitative attributes were assigned to a limited amount of intervals identified by progressive numbers. Such discretisation process [23] for each attribute *A*, generated a variable number *n *of partitions (*A_i_n_*; *i *= 1,.., *n*). The first partition *A_1_n _*included the lowest values of *A *and the last partition *A_n_n _*included highest values of *A*. Items (i.e. the attribute associated with a relevant discretised value) similar to the qualitative items could thus be generated (Figure 2).

Self organising maps (SOM) were used to cluster the values of the attributes. SOMs are widely known as a powerful clustering tool [24] and could overcome the disadvantages related to other unsupervised approaches as the *equal frequency intervals *or the *equal interval width *techniques. [25]. The latter methods, imposing an equal number of points belonging to each interval or, similarly, each interval having a pre-determined length, may generate meaningless or even empty intervals. SOMs were chosen and purposely implemented to properly isolate residual outliers [26] from the distribution of values of an attribute, to correctly define the clouds of values and to automatically set the optimal number of intervals [27]. Values and/or intervals were mapped in a discrete domain as integer numbers and items were created. In this way, the database became a set of itemsets.

The search of frequent itemsets was performed on the selected dataset. Itemsets with support greater than the *minimum support *were found. The set of itemsets that appeared frequently in the transactions of the database was then identified. This step of the process was the most demanding in terms of processing time and computer memory occupation.

The search for association rules was accomplished by using the APRIORI algorithm [10] which was shown to perform better then other common algorithms such as AIS [21] and SETM [28]. The APRIORI algorithm iterated the two following steps:

• building of a candidate set *Ck *of itemsets, counting their occurrences;

• defining "large itemsets" *Lk *on the basis of support constraints.

**Figure 3:**
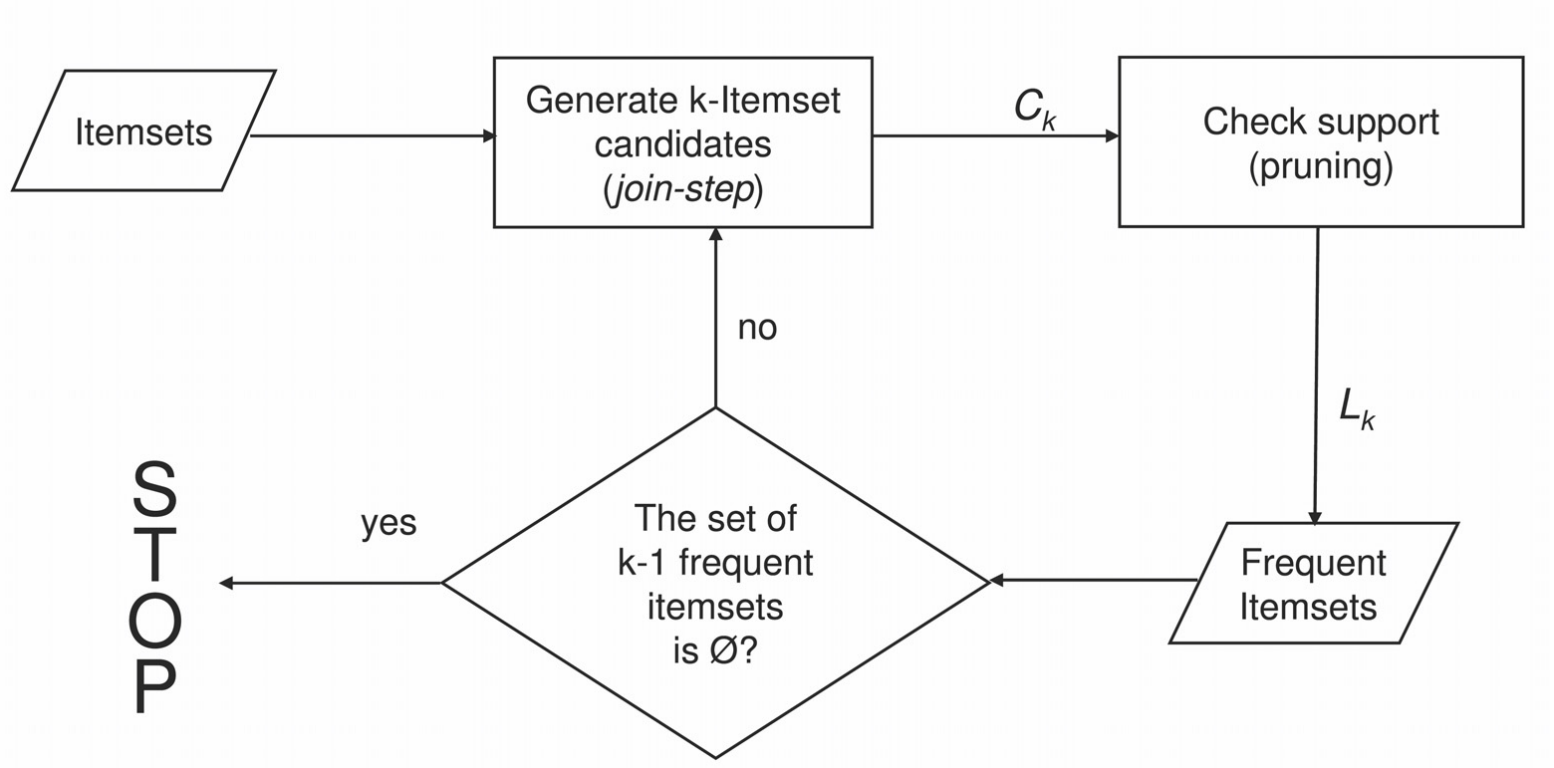
The Apriori algorithm applied to the database under analysis. The two phases of the Apriori algorithm are highlighted. The first, referred as "join step" phase, aimed at the generation of the candidate itemsets C_k _built starting from L_k-1_, the frequent itemset of the previous phase. In the second phase the C_k _itemsets underwent to a "pruning" procedure that selected the frequent itemsets L_k _on the base of the support check.

In figure 3, the main steps of the algorithm are illustrated.

Each frequent itemset generated a set of rules and each rule was scored by its confidence. Only rules whose confidence was higher than the *minimum confidence *reached the following phase. The selected association rules represented the knowledge extracted from the database expressed in a quasi natural language that the user could interpret. Efforts were made toward a clustered representation of the set of rules to increase readability and interpretability of information.

A software project for the data mining phase was purposely designed and implemented as follows: the software received as input all data from the database and returned a text file containing a list of the discovered association rules and all the possible unified rules and definitions derived from the entire dataset. Support and confidence thresholds were set to 35% and 85%, respectively. Considering that in a rule more than one consequent can be found [29], a maximum number of consequent items had to be set. In this analysis such number was experimentally set to 4 to avoid the presence of meaningless items in the resulting rules.

### Materials

Healthy adult volunteers (N = 110), both males and females between 22 and 87, participated in the study, executing a total of more than 1100 trials. They were initially asked to sit on a seat. The height of the seat was set at a value equal to the subject's tibial plateau height [30]. Subjects could choose the distance of their feet from the seat and had to keep them parallel at a distance equal to that measured between iliac anterior superior spines. Both footprints were then drawn on the floor, ensuring that the subject's feet were in the same position during all the trials. In addition, medio-lateral and antero-posterior coordinates of selected foot points were measured. Anthropometric parameters, such as the body mass and the length of the lower limb segments, were also obtained. Subjects were asked to rise from the seat at the preferred speed after an audio start signal and look at a frontal one metre distant fixed point at the height of 80% of their eyes' height, maintaining the orthostatic posture until the stop signal. Arms were kept crossed on the chest during the trial to avoid that arm swing could affect CM movements.

Ground reaction forces were measured using a six component Bertec force platform (0.4 m*0.6 m), positioned under both the seat and the subject's feet. Data were collected at a sampling rate of 100 Hz and pre-processed with an internally developed Labview^® ^software (National Instruments Inc.). First, force platform signals were digitally low-pass filtered (second order Butterworth filter 15 Hz cut-off frequency). Data were then fed into the TIP model, which yielded the kinematic and kinetic time functions (displacement, velocity, force/couple and power) of the LA and SA. FA variables were not analysed since their contribution to the motor strategy was considered negligible, given the sagittal symmetry of the STS motor task. From these functions a subset of kinematic and kinetic variables (KK-set) was extracted including time events of the task (normalised with respect to the duration of the whole task, see caption of Table 1 for the complete list of variables) and together with experimental set-up and subject specific parameters were stored in a Microsoft Access database, and loaded using a Windows ODBC interface [31]. The resulting database contained a total of more than 52,000 items. The number of analysed attributes was set to 47, as listed in Table 1.

**Table 1:** The 47 attributes analysed. They included subject initial conditions (ankle and thigh angles) and experimental setup/anthropometric parameters (seat height, thigh length, foot length, TIP1 hinge and malleoli coordinates), KK-set variables and important time instants. The KK-set was made of displacements (*Disp*), velocities (*Vel*), forces or couples and powers referred to the two LA and SA actuators. *So *referred to seat-off. In addition, ML, AP and V referred to the medio-lateral, antero-posterior and vertical directions. Finally, the attributes labelled with an initial "T" represented the instant of occurrence of the corresponding quantity (e.g. the attribute *MaxLAVelASO *referred to the maximum value of LA velocity after the seat-off and the attribute *TMaxLAVelASO *represented the corresponding instant of occurrence).

**Anthropometric and Experimental set-up Attributes**	**Kinematic and Kinetic Attributes**	**Time-Attributes**
*RightAnkleAngle*	*MaxSADispBSO*	*Duration*
*LeftAnkleAngle*	*MaxSAVelBSO*	
*APRightMalleolusCoord*	*MaxSACoupleBSO*	*TMaxSADispBSO*
*APLeft MalleolusCoord*	*MaxSAPowerBSO*	*TMaxSAVelBSO*
*APHingeCoord*	*SADispSo*	*TMaxSAForceBSO*
*MLRightMalleolusCoord*	*SAVelSo*	*TMaxSAPowerBSO*
*MLLeftMalleolusCoord*	*SACoupleSo*	
*MLHinge Coord*	*SAPowerSo*	*t*_*So*_
*VRightMalleolusCoord*	*MaxSADispASO*	
*VLeftMalleolusCoord*	*MaxSAVelASO*	*TMaxSADispASO*
*VHingeCoord*	*MaxSACoupleASO*	*TMaxSAVelASO*
*SeatHeight*	*MaxSAPowerASO*	*TMaxSAForceASO*
*ThighLength*	*MaxLADispASO*	*TMaxSAPowerASO*
*ShankLength*	*MaxLAVelASO*	*TMaxLADispASO*
*FootLength*	*MaxLAForceASO*	*TMaxLAVelASO*
*RightThighAngle*	*MaxLAPowerASO*	*TMaxLAForceASO*
*LeftThighAngle*		*TMaxLAPowerASO*

## Results

Various rules and definitions were found. Among them, some referred to obvious relationships such as those related to symmetry between right and left coordinates, others related a single item of a temporal parameter (*TMaxSADispASO_3_3_*) as a consequent of the following kinematic and kinetic items:

a) *MaxSADispBSO_3_6 _*and *MaxSAPowerBSO_1_6_*, before seat-off;

b) *SAVelSo_3_6_*, at seat-off;

c) *MaxLADispASO_3,4_6_*, *MaxLAForceASO_4_5_*, *MaxSADispASO_3_6 _*and *MaxSAVelASO_2,3_5_*, after seat-off;

and of the following time events:

*Duration_1_6_*, *TMaxSADispBSO_3_6_*, *TMaxSAVelBSO_3_6_*, *TMaxSACoupleBSO_2_6_*, *TMaxSAPowerBSO_2_6_*, *TMaxLADispASO_5_6 _*and *TMaxSAPowerASO_3_5_*.

The attribute *TMaxSADispASO *was the attribute with the lowest number of partitions (three partitions) and its last partition included about 90% of the observations.

**Figure 4:**
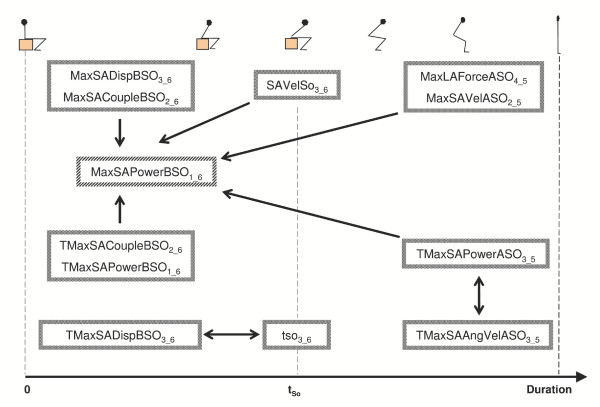
Graphic cluster representation of both the rules and the definitions found in the study. The first ones, marked with a single-ended arrow, were found to have a confidence ranging from 86% to 96%. The second ones, marked with a double-ended arrow, both presented a confidence of 95%. Involved items are positioned according to the STS time subdivision (BSO and ASO phases and seat-off timing).

The discovered definitions and rules that could not be easily predicted are illustrated in Figure 4 using a cluster representation, which highlights inner and crossed relationships among items of each phase of the task; values of confidence are reported in the figure caption.

The definitions related exclusively time instant items:

• *TMaxSADispBSO_3_6 _*←→ *t_so3_6 _*[95 %],

• *TMaxSAAngVelASO_3_5 _*←→ *TMaxSAPowerASO_3_5 _*[95 %],

The first definitions related the 'average' time of occurrence of maximum sagittal displacement during BSO (partition 3 of 6) to 'average' values of *t*_So _(partition 3 of 6). The second definition associated the time instant of maximum sagittal velocity to that of maximum power, both after the seat-off. Moreover, meaningful rules were found that involved as consequent the item *MaxSAPowerBSO_1_6_*. This item showed relationships, with a value of confidence varying between 86% and 96%, with the following kinematic and kinetic items:

*MaxSACoupleBSO_2_6_*, *MaxSADispBSO_3_6_*, *MaxLAForceASO_4_5_*, and *MaxSAVelASO_2_5_*; and the following temporal items:

*TMaxSACoupleBSO_2_6_*, *TMaxSAPowerBSO_1_6_*, *TMaxSAPowerASO_3_5 _*and *SAVelSo_3_6_*.

The partitions corresponding to the attributes involved in both rules and definitions, their support and their range of variability expressed in the relevant units of measurement (UoM), are reported in Table 2.

**Table 2:** Items involved in the discovered rules and definitions, their support and their range of values.

**Item**	**Support (%)**	**Range**		**UoM**
*Duration _1_6_*	45.0	1.01	1.61	s
*MaxSADispBSO_3_6_*	41.3	29	36	deg
*MaxSACoupleBSO_2_6_*	35.5	0.06	0.09	Nm kg^-1^m^-1^
*MaxSAPowerBSO_1_6_*	81.5	0.00	0.08	Wkg^-1^m^-1^
*SAVelSo_3_6_*	40.1	0.58	0.77	rad s^-1^
*MaxSADispASO_3_6_*	59.7	-3	14	deg
*MaxSAVelASO_2,3_5_*	84.7	0.44	1.32	rad s^-1^
*MaxLADispASO_3,4_6_*	87.2	45.4	54.5	% of TIP2 final length
*MaxLAForceASO_4_5_*	43.9	10.85	11.94	N kg^-1^
*TMaxSADispBSO_3_6_*	36.7	39.2	48.0	% of duration
*TMaxSAVelBSO_3_6_*	37.9	34.6	42.1	% of duration
*TMaxSACoupleBSO_2_6_*	47.5	10.3	15.1	% of duration
*TMaxSAPowerBSO_1,2_6_*	92.1	20.5	26.3	% of duration
*t_So3_6_*	35.4	46.9	55.9	% of duration
*TMaxSADispASO_3_3_*	89.2	87.1	99.9	% of duration
*TMaxSAVelASO_3_5_*	44.6	42.0	54	% of duration
*TMaxSAPowerASO_3_5_*	45.4	42.2	54.4	% of duration
*TMaxLADispASO_5_6_*	36.4	90.8	96.3	% of duration

The items reported in Table 2 belong to a subset of 18 attributes of the original 47. Only a limited number of items was involved in the discovered rules.

## Discussion

The data mining analysis allowed for the discovery of both definitions and rules relating various items obtained from a MMIM analysis of the STS motor task. The most obvious and/or expected relationships, such as those related to the symmetry between right and left coordinates, also noticeable by a visual examination of the task as performed by the investigated subject, were included in the set of discovered rules and definitions. The finding of such relationships provided elements to confirm the validity of the data mining analysis. The set of rules found that related the temporal item *TMaxSADispASO_3_3 _*to various temporal, kinematic and kinetic items needs a further analysis to be interpreted. In fact, the attribute *TMaxSADispASO *was mapped in only three partitions and most of its observations were concentrated in the last partition. This circumstance rendered highly probable the presence of rules relating the item *TMaxSADispASO_3_3 _*to those items of the various attributes with support higher than 35%. Therefore, these rules were used to highlight items involved with a considerable support and therefore the usefulness of such rules was deemed limited. In general, when interpreting a rule/definition found, the analyst should be aware not only of both its confidence and the support of the items forming it, but also of the number of partitions in which the attributes involved in the rule/definition were divided. The fewer are the partitions used for a quantitative attribute, the higher is the probability of finding rules/definitions unsuitable for drawing specific patterns. This is particularly true when most of the observations fall in a single partition of the attribute. Conversely, some of the rules and definitions discovered by the data mining analysis highlighted relationships that could not be easily predicted otherwise. The two definitions reported in the results section, that related time instant items, indicated that specific 'average' timings (items belonging to central partitions of the corresponding attribute) of the sit-stand task were closely related. This finding is consistent with those present in the literature [32,33]. In particular referring to the second definition reported, since power is the product of moment and angular velocity, the definition that associates 'average' values of the instant of maximum sagittal velocity to those of maximum power after the seat-off could be predicted.

Very interestingly, the relationships of the item *MaxSAPowerBSO_1_6 _*with several KK-set items showed the importance of the SA in the execution of the task. In fact, almost all rules relating KK-set items to the *MaxSAPowerBSO_1_6 _*regarded the sagittal actuator. When the maximum SA power during BSO occurred early in the task, its value was among the lowest (*TMaxSAPowerBSO_1_6 _*→ *MaxSAPowerBSO_1_6_*). Low values of SA maximum power during BSO also occurred in combination with low-to-medium couple values (*MaxSACoupleBSO_2_6 _*→ *MaxSAPowerBSO_1_6_*) and early in the phase (*TMaxSACoupleBSO_2_6 _*→ *MaxSAPowerBSO_1_6_*). The latter rules showed that before seat-off kinetic variables of the main actuator are strongly related to each other and their timing. Given a value of one of them, a limited range of values is to be expected for the others. Moreover, 'average' SA velocity at seat-off was found to be present in combination with low maximum SA power at BSO (*SAVelSo_3_6 _*→ *MaxSAPowerBSO_1_6_*) showing that relatively high speeds at seat-off could be reached even when the power exerted before seat-off was low. The presence of low value partitions in the rules may suggest that most healthy adults tend to use the least amount of energy necessary to complete the first phase of the task, showing an effective strategy of reduction of the energy expenditure [34]. A validation of this hypothesis could be obtained in a rehabilitative context, by studying databases containing data of samples of different populations (i.e. healthy subjects versus subjects with a specific motor functional limitation).

The rules relating the low maximum SA power during BSO to variables occurring during ASO allowed for BSO-ASO crossed inferences. When medium-to-high maximum LA force during the elevation of the centre of mass toward the standing position was found, a low maximum SA power was generated by the SA before seat-off (*MaxLAForceASO_4_6 _*→ *MaxSAPowerBSO_1_6_*). Moreover, consistent with the relationship to the SA velocity at seat-off, low maximum power of the SA during BSO occurred in combination with low-to-medium maximum velocity values during ASO (*MaxSAVelASO_2_6 _*→ *MaxSAPowerBSO_1_6_*) showing that after seat-off a low-to-medium SA velocity can be reached and kept during the remaining part of the task, even when a low power is exerted before seat-off. Finally, average timing of occurrence of maximum SA power after seat-off implied a low maximum SA power before seat-off (*TMaxSAPowerASO_3_5 _*→ *MaxSAPowerBSO_1_6_*) showing that, when the task is performed with an 'average' distribution of the time instants, the power exerted before seat-off is at its lowest values.

The results' representation of Figure 4 could be used as the main outcome of the knowledge discovery process to be used by the analyst as a reference for the examined population. In the case of the present study, the patterns found are representative of the most common characteristics of the way healthy adults, of both genders and in a wide age range, perform the sit-to-stand task. Any deviation from these patterns found in a healthy adult could be considered as an uncommon characteristic. The patterns resulting from the analysis of a database containing a subgroup of the subjects examined in the present study (i.e. female subjects or subjects over the age of 65) could be considered as specific of the selected subgroup. Similarly, if the analysis is applied to a database of subjects affected by a specific pathology then the resulting patterns would characterise that population of subjects. The comparison of those patterns and the patterns found in the present study would highlight how differently the two groups perform the task. In perspective, from a rehabilitation standpoint, the output of data mining analyses applied to various groups of subjects performing various tasks could be used as a reference tool to evaluate the performance of subject under examination and, therefore, her/his level of mobility.

## Conclusions

The study focused on finding the most frequent patterns of biomechanical variables and parameters obtained from dynamometric measurements of healthy subjects performing the sit-to-stand motor task. Data collected in a large database underwent a knowledge discovery process. The size of the database is strongly related to the simplicity of the data acquisition procedures. Simple and less expensive experimental set-ups allow the gathering of more data and in more locations than high-cost experimental set-ups and procedures. Data acquired from force platforms, processed with specific biomechanical models, represent a favourable condition to apply knowledge discovery processes effectively. In this study, data from volunteers in a large age range and of both genders were analysed in order to extract the most common patterns of healthy people performing the task. The results of the knowledge discovery process showed that sit-to-stand time events were strongly interdependent. Low maximum sagittal power values before seat-off were strongly related to numerous parameters both before and after seat-off, highlighting, among other characteristics, that most often a low power before seat-off is related to a regular occurrence of time instants and to low-to-medium sagittal speed from seat-off to the end of the task. The patterns found may be considered as typical rules of the sit-to-stand motor task and could constitute the basis for comparisons of patterns characteristic of different groups. The knowledge acquired in this study is the first step in the direction of developing a robust clinical tool to evaluate subject mobility.
